# Motivations and reasons for women attending a Breast Self-Examination training program: A qualitative study

**DOI:** 10.1186/1472-6874-10-23

**Published:** 2010-07-10

**Authors:** Rea-Jeng Yang, Lian-Hua Huang, Yeu-Sheng Hsieh, Ue-Lin Chung, Chiun-Sheng Huang, Herng-Dar Bih

**Affiliations:** 1 Department of Nursing, National Taipei University of Nursing and Health, 365, Mind Te Road Taipei 112, Taiwan; 2 School of Nursing, National Taiwan University, 1, Sec. 1, Jen-Ai, Road, Taipei 100, Taiwan; 3 Department of Agricultural Extension, National Taiwan University, 1 Sec. 4, Roosevelt Road Taipei 106, Taiwan; 4 National Taiwan University Hospital and National Taiwan University College of Medicine, 1, Sec. 1, Jen-Ai, Road, Taipei 100, Taiwan; 5 Graduate Institute of Building and Planning, National Taiwan University, 1 Sec. 4, Roosevelt Road Taipei 106, Taiwan

## Abstract

**Background:**

Breast cancer is a major threat to Taiwanese women's health. Despite the controversy surrounding the effectiveness of breast self-examination (BSE) in reducing mortality, BSE is still advocated by some health departments. The aim of the study is to provide information about how women decide to practice BSE and their experiences through the training process. Sixty-six women aged 27-50 were recruited.

**Methods:**

A descriptive study was conducted using small group and individual in-depth interviews to collect data, and using thematic analysis and constant comparison techniques for data analysis.

**Results:**

It was found that a sense of self-security became an important motivator for entering BSE training. The satisfaction in obtaining a sense of self-security emerged as the central theme. Furthermore, a ladder motivation model was developed to explain the participants' motivations for entering BSE training. The patterns of motivation include opportunity taking, clarifying confusion, maintaining health, and illness monitoring, which were connected with the risk perception for breast cancer.

**Conclusions:**

We recognize that the way women decide to attend BSE training is influenced by personal and social factors. Understanding the different risk assessments women rely on in making their health decisions is essential. This study will assist researchers and health professionals to gain a better understanding of alternative ways to deal with breast health, and not to be limited by the recommendations of the health authorities.

## Background

As a means for early detection of breast cancer, breast self-examination (BSE) has shown that cancer may be discovered at anytime. Most women accept the idea that breast cancer may happen to any of them, yet at the same time, some of them fear discovering the disease. Although researchers have studied the determinants of BSE, there has been relatively little understanding about the motivation of women to learn breast self-examination.

A more in-depth exploration of women's experiences in relation to BSE might provide an insight into their behavioral choices. Therefore, we have attempted to explore the reasons why women want to learn BSE skills, as well as explore and illustrate the patterns of how women make the decision to learn this technique. The research questions involved were 2-fold: (a) what are the overall approaches that women decide to enter a BSE training program? (b) what are the personal and social factors related to the motivation for making the decision? For this study, an overall approach was defined as a general way in which a person cognitively addresses a given decision-making task.

Baxter [1] announced that women no longer need to examine their breasts. This has influenced the policies of some medical organizations, such as the American Cancer Society (ACS). For years, the ACS recommended that BSE conducted on a monthly basis was an important means of early cancer detection. Now the ACS promotes mammography for routine cancer screening rather than BSE [2]. Women's decision making about BSE was complicated by being provided with paradoxical advice from health professionals or other sources of information. For example, the guidelines from the Department of Health in Taiwan encouraged women to do BSE monthly, but the media and physicians only encouraged women to participate in an annual examination in the hospital with clinical palpation or mammography, and purposely told their clients that the monthly BSE might be useless. These inconsistent guidelines increased the uncertainty of women making a decision regarding BSE.

Motivation is defined as an internal state or condition that serves to activate or energize behavior [3]. For example, seeking a sense of security often arouses the inner drive into an action of decision-making. In the healthcare field, steps involved in making a decision may be remembered using the mnemonic BRAND, which includes benefits of the action; risks in the action; alternatives to the prospective action; nothing: that is, doing nothing at all; and decision [4]. These steps also explained how the surrounding sources or information from the media and physicians could make such an impact in women's attitude to BSE.

BSE is represented as an opportunity for having some control in the face of a potentially life-threatening disorder. Vahabi and Gastaldo [5] noted that the logical behavior based on our current understanding of prevention is one of a philosophy of risk that incorporates a secularized approach to life where events do not simply happen without warning, but can be predicted, i.e. individuals should plan for the future and take judicious steps to ensure protection against misfortune, whilst retaining responsibility for their affairs.

Thus, the underlying assumption behind this philosophy is that women must have knowledge of their potential and covert possibility of breast cancer in order to protect themselves. According to Rothman and Kiviniemi [5], people's knowledge about health risks and the benefits of taking protective action is the driving force in their decision making. By extension, efforts directed at appraising people's decisions consider that human beings are rational, with a fundamental goal of maintaining or enhancing their health.

There has been little research published on how women make decisions regarding BSE. Most researchers have focused on factors associated with women's behavior related to BSE rather than on the decision-making process that preceded the behavior. For example, the low ratio of women who practice BSE monthly has been attributed to several other factors, such as inadequate knowledge about the risk of breast cancer and the life-saving advantages of BSE [6-8]. The concept of rationality in decision-making is prevalent in substantive theories of rationality, such as the Health Belief Model (HBM). However, the HBM does not adequately give an explanation for the low rate of BSE practice [9]. The reason for this is partly due to the fact that many studies have found little variation in the model's independent variables, i.e. most women believe that the threat of breast cancer can be high and the benefit of BSE is great. But this knowledge itself is not enough to ensure frequent and/or competent BSE practice. The inadequacy of cognitive-behavioral models to explain women's practice of BSE and the need to consider the social context in different cultures and groups have previously been recognized [9,10]. However, this research did not examine how women actually took these factors into account when they considered performing or not performing BSE.

The above literature highlights the fact that BSE is a complex issue. It is not only a medical one, but a sociological one. In summary, much of the research in the field of BSE practice has focused on factors associated with BSE frequency using standardized questionnaires. Thus, ascertaining how women make decisions regarding BSE urgently requires more empirical study.

## Methods

### Participants

This study was approved by the Institutional Review Board at National Taiwan University. The researcher obtained both written and oral informed consent from the participants. The paper constitutes part of the result of the BSE training project. To participate in this study, women were required to be < 50 years old, not previously diagnosed with breast cancer, and significantly from a high-risk and under-protected population among Taiwanese women. The mortality rate from breast cancer in women of this age group has been gradually increasing and yet they do not have access to free mammography. BSE therefore is potentially an easily available and accessible method for them.

The researchers recruited participants who had a better knowledge and experience with the subject being studied. A purposeful sample was recruited for in-depth interviews. Interviewees had participated in a large (n = 203) community-wide BSE training project designed to increase BSE competency. This research has also shown that studying different age groups and education levels of women can bring greater variety to the results. Thus, recruiters were asked to select women participants from various categories, in age and education levels. The recruiters met to choose potential research participants and then the field researcher asked the selected women if they would participate. The number of interviewees was based on the principles of saturation. Since the qualified participants were collected while specifying with the 203 participants along with the interview and analyze. Therefore, the final number of participants was not pre-decided. After 28th group interview, it was hard to elicit new nuances from the interviews. It was considered that the investigation had reached a preliminary saturation point, and the collection of data was discontinued after the 31st group interview had finished. This final sample comprised 66 women aged between 27-50 years old. Most (87.6%) were married. Sixty-four women (96.9%) had at least a high school level of education. Eleven participants (16.7%) reported that they had a first-degree family history of breast cancer. About 35.6% of the participants had previously performed BSE before the training program, but only 3 women had been practicing BSE monthly. There were 22 women (33.3%) practicing BSE monthly after the training program.

### Data collection

Interviews were held in the community meeting rooms, at the participant's or the researcher's home. The interviews took 30-120 min to complete and were audio taped. Of the 31 interviews conducted, 28 were used with a small group of 2-3 women, and 3 were conducted as one-on-one interviews. Some positive evidence revealed that data regarding these perceptions may be enriched in a group setting, where individual participation can be enhanced through group interaction [11]. One-on-one interviews were conducted simultaneously to examine the illicit data for comparison. The only field researcher, an instructor in the field of women's health and physical examination in the nursing school, also facilitated interviews in this study. Before interview, these participants have met with the researcher twice in order to conduct the BSE training and examine their BSE competency after 4 months. The intention was therefore to facilitate rapport and explain that the interview was a reciprocal process. Participants were open to discuss their experiences, ask questions, and request clarification regarding the process.

Participants began the interview by describing their reasons, decisions and motivations for entering the BSE training program, which had been launched in the community 4 months earlier by the field researcher. The women were encouraged to frame the issue of BSE broadly, and generate some topics for discussion which included (see AppendixI): (a) their reasons for deciding to attend the BSE training program; (b) experiences of their BSE at home; (c) their feelings while examining their own breasts; (d) favorable circumstances for their monthly BSE practices; (e) promising to practice BSE frequently thereafter and the compelling reasons for this; (f) sharing stories about their relatives, friends, and acquaintances with breast cancer; and (g) thinking about the risk of getting breast cancer. The same semi-structural questions were asked with the individuals and small groups.

### Data analysis

Thematic analysis and constant comparison techniques were employed. Thematic analysis, data analysis and the interpretation of data were facilitated using Benner's [12] paradigm cases. In addition, specific guidelines, such as comprehending, synthesizing, theorizing, and re-conceptualizing as described by Morse [13] were used. Data interpretation commenced with a verbatim transcription of each of the 31 audio-taped interviews. Each interview was read thoroughly in its entirety. The field researcher "stepped back" and reflected on each interview as a whole in order to grasp the uniqueness of each woman's description of their experience of BSE.

The transcripts were then examined in detail to identify basic patterns and recurrent themes using line-by-line coding to examine, compare and begin developing conceptual categories. Categories were developed inductively using the constant comparison method [14]. This method of categorization demonstrates the expectation on the comparison of the variety between different results, and clarifies the similarities or differences in each item to create an identifiable key theme for the research. All coding was completed by the field researcher. To enhance the accuracy of the account of our research, after completing data analysis, as a form of member-checking [15], the concepts were given to 5 research participants for their confirmation, and to discuss the extent to which the important themes that we had identified using our analysis of the interview transcripts reflected their subjective experience.

## Results

**Figure 1:**
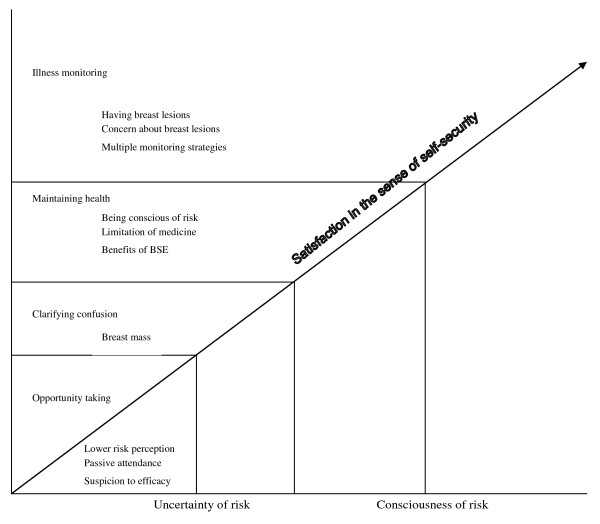


How do women face the threat of breast cancer? Our findings indicated that women's participation in BSE training was a purposeful action. Personal safety needs provided them with the principal motivation, i.e. a sense of self-security emerged as the central theme of participants' efforts to practice BSE. Each woman had her own personal reasons for participating in the BSE training. However, the common motivation related to how women assessed their risk of breast cancer, as influenced by individual and social factors. According to the level of the women's risk perception, a ladder of motivation was found in the results. The 4 patterns of motivation were identified as: taking opportunities, clarifying confusion, maintaining health, and illness monitoring. The 4-ladder motivation model and its intertwined dimensions are shown in Figure 1. The categories are further expanded, and all names used in the analysis are pseudonyms.

### Satisfaction in the sense of self-security

The results indicated that almost all of the women could not deny the possibility of having breast cancer, but living in a conservative society like Asia, most women deal with their worries on their own. Fon Shin (in Chinese), the description of storing your heart in a safe-keeping place carries a similar meaning to a sense of self-security in English. Fon Shin has also become the barrier in women with possible breast cancer to look for professional help as their bodies cannot be freely exposed to a stranger's eye. One woman explained *"I wanted to learn BSE so I can do self-examination without worries" (A28)*. A second woman said *"I think if I learn the skills I can do it more often by myself, with my own feelings on them so that my mind can be released" (C56)*. In as much as women's existence is threatened by breast cancer, a self-examination procedure needs to be mobilized to give a sense of self-security. The study showed that satisfaction in a sense of self-security was related to autonomous decision making and action; as another participant explained, *"I want to understand my body more" (D10)*. Therefore, in general women who understand how to self-examine and understand their own body condition could be important in the prevention of possible lesions.

PS: In parenthesis, the first letter labeled the level of ladder: A, opportunity taking; B, confusion clarifying; C, health maintaining; and D, illness monitoring. The second letter represented the number of cases. Ex. (A28) it refers the quotation from the 28 case in the opportunity taking ladder.

### Ladder of motivation

Our findings indicate that the salient motivations for entering the program could be identified as: taking opportunities, clarifying confusion, maintaining health, and illness monitoring. These were connected with risk perception of breast cancer and were not isolated from one another. Indeed, they move back and forth dynamically, depending on individual and social conditions. Forty out of 66 women felt that health maintenance was the major motivation for their attending the project, whereas 9 participants said that they attended for illness monitoring, 2 for confusion clarifying, and 15 for opportunity taking.

#### 1. Opportunity taking

Some women claimed that they were taking advantage of an opportunity, and their participation in BSE was not solely based on medical considerations. Opportunity taking was expressed in 3 categories, as a lower risk perception, as suspicious to the efficacy of BSE, and as a passive or incidental attendance at the program.

##### 1-1 Lower risk perception

This concept is reflected in the women's discussion about the low prevalence of breast cancer among their family's members and friends. Fifteen participants intimated that they had no family history of cancer and regarded themselves as women with a lower risk, compared with the average woman. Furthermore, after comparing themselves to those stereotypical images about physical condition and lifestyle, they positioned themselves at a lower risk.

"What risk? I do not feel it is high for me because I neither have genetic inheritance nor those so-called cysts. As I do not have large breasts, I am not the target." (A46)

"I am sort of mentally fluky, it derived from not having a family history of breast cancer and I have a healthy lifestyle, no smoking, no drinking; I also do exercise so, I believe that I am not at high risk." (A28)

"Breast cancer, I feel it is impossible to occur to me (laugh). I am married and have given birth to children. I do not feel any changes or problems with discharge, and no pain." (A15)

However, one woman, whose aunt (her mother's sister) had breast cancer still did not associate herself with high risk. She was able to reconcile herself with perceptions of being at a lower risk. Maybe she created an alternative explanation about the etiology of breast cancer. She stated that: *"Although my mother's sister had breast cancer, I have a faith, though a bit strange, that l probably will not get breast cancer. Furthermore, I watch my diet, and eat a lot of vegetables and fruit while avoiding meat and greasy foods." (A23)*

As stated above, women mostly thought that their health is at low risk based on their medical knowledge and beliefs instead of observing changes in their own body.

##### 1-2 Suspicion to efficacy

In this group, the participants did not perform BSE in the 6 months prior to BSE training. Some had used other screening methods and had been informed of no abnormal findings. However, a few women were suspicious, to the extent that they examined their breasts themselves.

"I have had done it twice by sonogram and been palpated by the doctor 2-3 years ago prior to the program. Anyway, only a doctor can conduct an efficient breast examination. I did not think that I could do so by myself. I attended this program since I wanted to know some of the skills for palpating breasts. I know that I have touched the bulk in my breast, and it made me nervous while I touched it." (A48)

"It is my emotional obstruction, some of my friends said that it's ok enough to be checked out by physicians. They believe in an expert, not in themselves. I wondered at their inactivity to the learning resources...." (A46)

"I doubted I could learn it well." (A21)

##### 1-3 Passive/incidental attendance

These women stated that their participation in the program was influenced by external forces, such as those from ties, by friends and the media. For example:

"I met Miss Lin [the assistant in this research], who invited me to join in your BSE project. I thought it is the age that I have to care about this disease. Generally, I did not pay attention to it. I was passive and just came to try it." (A15)

"At first, I attended and supported the program just for taking my responsibility as a mother-volunteer in the school. I am not really interested in learning BSE." (A21)

"In fact, I am not a health believer; I do not take care much about myself. Honestly, I thought this is a rare chance to learn something about breast examination. I entered the program just passively. Particularly, at the time when Mrs. Kuo [the richest entrepreneur in Taiwan] died of breast cancer, everyone was talking about it. It made me panic." (A28)

"It was an important incentive that I could learn along with my peers." (A28)

"I joined the training program because of being pushed by my friends." (A15, A33)

The data showed that women, who had a better chance of accessing some BSE programs or information, and especially to someone who is trustworthy, would gain more incentive to participate in the examination.

Low risk perception, suspicion to efficacy, and passive/incidental attendance are the reasons for those women in the program who still hold some uncertainties toward their bodies, and explain their concerns about joining the program.

#### 2. Clarifying confusion

Misunderstandings concerning the concept of breast mass have made participants confused about being at a higher risk.

##### Breast mass

Two participants with no family history of breast cancer found buds inside their breasts. They had previously practiced BSE, but had not had a mammography or a sonogram before the training program. They had questions about breast traits, and did not know the characteristics of potential signs of breast cancer. They were panic about the mass existing in their breast.

"I heard that breast cancer might be found through lumps in advance. When I touched my breasts, I felt the mass in them. The mass is still there but there have not been any changes up to now. I think that techniques of BSE can be of help for self-examination." (B11)

"I always palpated some masses in my whole breasts. They felt like in pieces. I wondered what those were. Are they normal or abnormal? Then I went to visit a doctor. The surgeon did not tell me anything (laugh). I asked for an examination, he replied he did not like to do a clinical breast exam. I thought maybe there was some difficulty in a male doctor palpating woman' breasts, although I am ok about it, he may be not. Then what? I do not know where I can ask for help. You know, nowadays there are few programs of breast self-examination for women." (B20)

For women who wanted to increase their knowledge concerning breast cancer and avoid further confusion, they approached the BSE scheme when they found a breast mass and were unable to get answers by themselves or especially from professional doctors about their concerns.

#### 3. Maintaining health

Of the 40 participants in this group, 17 women had practiced BSE at least once during the past 6 months, and 12 women had also previously had a mammography or sonogram before the training program. They claimed that they wanted to learn BSE for health protection as an early detection method. The concept was constructed in 3 dimensions, first perceiving the risk of breast cancer, second realizing the limitations of medicine, and third knowing the benefits of breast self-examination.

##### 3-1 Being conscious of risk

In this group, all women expressed that they perceived the risk of breast cancer. However, the participants varied in assessing their risk of breast cancer. Seven women believed that their risks were connected with having a family history of breast cancer.

"My mother was diagnosed with a breast cancer last month." (C58)

"I am a person with a high-risk because my mother and my elder sister had breast cancer." (C34)

"I was nervous about having breast cancer because my mother had breast cancer, and had a mastectomy for one of her breasts last year. Also, I have used hormone replacement therapy." (C56)

A negative family history does make a woman more concerned about their breasts. Many women expressed that their being conscious of the risk of breast cancer was influenced by their friends or relatives having breast cancer.

"Because those 2 relatives [my aunt and my sister-in-law] and a friend have breast cancer, I feel this is a crisis in a woman's life." (C49)

"My neighbor next-door and a friend are having chemical treatments for breast cancer. I believe that women need to learn some skills of breast self-examination for early treatment. "(C5)

"I am surprised that one of my colleagues, who [28 years old] is younger than me, was diagnosed with breast cancer. She was so young. That is why I want to learn the skills..." (C8)

The perception of risk was irrespective of their age and more concerned with their understanding. The risk assessment was based on their assumptions about the characteristics of women.

"As a 47-year-old woman receiving hormone replacement therapy, this is time for me to take care of my breasts." (C62)

"Although I am young [27 years], I cannot exclude the risk of breast cancer since I am a woman." (C7)

"I found that women can have cancer at younger age. Yes, we have to protect our health from a young age." (C8)

"In the beginning, I attended this training due to my large breasts. I have heard that enormous breasts increase the risk of breast cancer. My mother's breast cancer, which was diagnosed last month after I learned the BSE, made me pay more attention to it." (C58)

The risk assessment was also influenced by social environment.

"Recently, breast cancer has been big news in the media since Mrs. Kuo - the richest entrepreneur's wife - died of breast cancer. That made me concerned about the breast self-examination. I want to learn how to do it." (C41, C40)

Therefore, we understood that women's conceptions of risk in breast cancer are constituted mostly by multiple sources such as past experiences of family members, relatives, friends, or others.

##### 3-2 Limitations of medicine

The dominant screening modalities of breast cancer are mammography, sonogram, and clinical breast examination. Participants have realized the limitations of medicine and technology, as the following excerpts illustrate:

"I feel that it is necessary to have a mammography, but I also have heard that mammography has its limitations, and the accuracy is not high. My friend had been in stage-II breast cancer, but it had not been detected by mammography, which was adopted one year and a half ago." (C35)

"I had a breast examination by a doctor, but he took less than 3 minutes." (C17)

"I felt uncomfortable when the doctor was doing a breast examination for me, because his facial expressions made me feel disgusted. I do not want to see that rotten doctor any longer." (C58)

"I felt that the doctor did a breast examination for me lacked concern. He did not do it under standardized procedure. In general, the doctors depend on the sonogram as the principal screening equipment. Mrs. Kuo died of breast cancer, although she had had regular mammography and sonograms." (C5)

"I was in hormone therapy and the doctor conducted a mammography for me. It showed no problem, but I am still so nervous when asking for a sonogram examination." (C65)

As we have revealed more disadvantages in current medicine practice, so the lack of humanity in medical practice has become a vital issue for the women.

##### 3-3 Benefits of BSE

Regarding to the perceived benefits of BSE as a protective strategy, most participants in this group know well that BSE can be helpful in finding the breast problem because their relatives and friends found the breast cancer by self-examination. BSE evidently works, as the following examples state:

"My friend discovered her breast cancer by touching her own body. Unfortunately, her tumor had been growing. If it had been found earlier, everything could be different. I think that every woman should do breast self-examination." (C35)

"The Vietnamese care-giver found the lump in my mother's breast, when she bathed my mother. She really did my mother a great favor. ...This is a good chance for me to learn these skills. I think that I can do it more often with my own fingers so that it might keep my mind at ease." (C56)

"I want to say I did BSE just for health, not intending to argue with the size of my breasts. The incentive of health is still strong (laughs). By the way, I have recommended the skills to my friends. For example, I told my boy-friend that I could teach his sister; as we are all women, we have to protect ourselves..." (C7)

Regarding breast cancer, even though most women never found any abnormal signs within their bodies, BSE can also eliminate the uncertainties of the disease, but learning BSE is also good for the health.

#### 4. Illness monitoring

In this group, all participants (9 women) had personal experience of abnormal clinical findings that were subsequently were diagnosed as benign. Although they did not report having a family history of breast cancer, having a breast with a benign lesion influenced their risk assessment. These participants considered themselves as being at higher than average risk. Illness monitoring became a motivation to their practicing BSE. The concept was composed of 3 elements: having breasts lesions, being concerned about breast lesions, and applying multiple monitoring strategies.

##### 4-1 Having breast lesions

Some women had experienced a benign lump caused by a fibro-cyst or fibro-adenoma, as well as nipple inversion and secretion.

"The mass in my breast was found by myself 10 years ago. The doctor believed that it was a fibrocystic problem." (D1)

"Last year, I felt my breast was tender, and then I discovered a mass. Yes, it was almost 2.5 cm large. The diagnosis showed it was a fibro-cyst." (D3)

"I had surgery for removing a fibro-cyst from my breast about 10 years ago." (D31)

"Last year, nipple inversions occurred in both breasts. In addition, I had seen little milk-like secretions on my nipples for many years ago. My doctor noticed and said that it did not seem to be a good condition and I have to follow up and take care." (D66)

From the above statements, we learn that women, especially those who have previously had a breast lesion, became motivated to monitor the condition of the breast regularly.

##### 4-2 Concern about breast lesions

Although the breast lesions of some participants were identified as benign, they were worried that the lesions would change from benign to malignant. Their considerations were associated with their knowledge.

"I have seen rotten breasts from pictures. Cancer can ulcerate the entire breast, how awful it is! You know, I am really worrying that I will get breast cancer and it will be too late to treat it. I am really worrying..." (D66)

"I was diagnosed with a fibro-cyst. I was covered with a cloud of shadow as I read the report which said it would change from being benign to malignant in 5-10 years." (D1)

##### 4-3 Multiple monitoring strategies

Women expect regular breast screening to be an essential procedure in saving their lives against breast cancer, but obviously not all women believe that any single-modality can achieve this result. To supplement any potential missing accuracy, some women have used more than one screening technique to ensure their health. Multiple monitoring strategies have been reported. In this group, 7 out of 9 participants have practiced BSE, and 5 women had done BSE at least once during the last 6 months. Furthermore, 8 women have previously received other screenings, such as mammography, sonogram, and clinical breast examination. The following are examples of their statements:

"I have been getting a mammography and sonograms annually now for > 10 years. I am so afraid of getting cancer. What can I do? I heard that the machines [mammography and sonogram] do not always work correctly. I happened to know there was a training program of breast self-examination, so I attended immediately. Feeling my breasts with my fingers seems better than seeing a doctor, who spends < 2 minutes for an examination." (D66)

"I used to have sonograms and also physicians palpation my breasts. In addition to the medical examinations, I also added an item for breast tumor marker (CA153). It makes me less fearful since I have a complete screening. You know, I touched and found a lump in my breast several years ago. Through self examination, I can observe the change of my breasts and this helps me to understand my own physical condition." (D1)

The study indicated that women's awareness of the threat of breast cancer or an abnormal breast finding, which caused them stress, would lead them to adopt coping strategies. The coping strategies are the learned behaviors of BSE, CBE (clinical breast-examination), and mammography. Many participants have used more than one modality to monitor their breasts. Self-examination for breast cancer is one of the methods of early detection which helps women to understand their own body, along with information about what is happening to their breasts.

## Discussion

BSE as a detector of breast cancer may save lives or be a trigger for fear and terror. What are the conditions that affect a women's decision-making? This study revealed that the motivation of all participants for entering BSE training was categorized into four patterns: taking opportunities, clarifying confusion, maintaining health, and illness monitoring. The ladder could be used to describe the risk perception that women feel at different levels. The greater the risk perception, the higher up the ladder motivation moved. The 4 motivation patterns have become interwoven with personal and social factors.

### 1. Personal factors influencing entry to BSE training

Women in this category believed in the importance of self-care and expected to take control of their own health condition. Referring to Haselton's statement in adaptive rationality, he suggests that the mind is remarkably well designed to deal with the important problems of survival and reproduction, and is not fundamentally irrational [16]. Furthermore, 3 personal factors were found to explain a women's motivation for starting BSE.

#### 1-1 The value of BSE

Leslie et al. [17] suggested that if women agreed that breast cancer could be detected through BSE, they would seek assistance to improve their techniques and be more likely to perform BSE [18]. The value of BSE is confused because of the conflict between government promotion and the struggle of medical benefit in practice. However, the advice from medical professionals to patients, and taking the social factors into consideration for women in making the decision to overcome the unwillingness to uncover her own body to strangers, have been the main controversy in this conservative society. Whether BSE could be successfully promoted to help women perform early self-detection examinations was complicated by too many external effects, instead of looking more seriously at women's feelings and the efficacy of BSE. We now understand, even with all the external effects and internal suffering with negative feelings, why women are still willing to search for a better way to deal with the risk of breast cancer in order to achieve a sense of security.

#### 1-2 Perception of risk

This study showed that almost all the participants wanted to learn breast self-examination, especially those who had symptoms or a family history of breast cancer. Choi [19] noted that the perception of susceptibility and severity could produce motivation sufficient to effect a behavior change. The perception of risk described by women in this study was based on their own experiences with abnormal breast symptoms and also how this was affected by their family members and friends. Previous studies gave similar results, a positive association between having an abnormal breast symptom [20,21], and a maternal relative with breast cancer [17]. Not surprisingly, these women were usually extremely anxious about the chances of getting breast cancer. They also expressed higher levels of unease in other aspects of their lives. Such women might be conscious about doing self-examination.

In this study, 15 out of 66 women thought that they were at a lower breast cancer risk than average. One woman claimed that she knew the characteristics of breast symptoms that were potential signs of breast cancer. Since she assessed her symptoms were without those characteristics, she concluded that it was not a sign of breast cancer and it did not threaten her health. Katapodi *et al. *[22] suggested that perceptions of being at a lower risk demonstrate an "optimistic bias" -- a state of mind of putting your "head-in-the-sand." However, some of the participants had received screening via mammography and sonogram prior to entering this BSE training program. It was noted that they still could not shut out the risks of breast cancer.

#### 1-3 Desire to have control over one's body

Some women indicated that they experienced confused feelings when they did a BSE. They neither understood how to do the examination, nor could they distinguish normal tissues from potentially dangerous lumps. Even though they had sought clarification from medical professionals, they remained unclear following their doctor's explanations. Therefore, the participants wanted to perfect their skills in performing a BSE so as to rely on their own evaluations. Some of these women even undertook a further strategy of multiple-monitors. They combined BSE with a mammography and sonogram to ensure greater reliability.

Consistent with our findings, another study has revealed that women constructed their own personal meanings about the benefits and limitations of BSE, as well as their use of this self-care behavior within their daily lives [23]. They used BSE as a means of gaining control over their fear of the threat of breast cancer. Previously scholars [24] had noted that following a potentially life-threatening physical illness, a new awareness of the body was formed and illness was perceived to be the catalyst for a positive behavior transformation. Salick and Auerbach [25] also found that the reclaiming of the physical body was a vital component to the stress-related growth process.

Summing up the reasons, we found that women may decide to enter BSE training as a coping strategy for dealing with the stress of perceived risk of mammary cancer. The ultimate outcome relevant to breast health behavior they desired was for breast health or the early detection of cancer. Results revealed that the sense of self-security became a great motivator.

### 2. Social factors influencing the practice of BSE

The decision to participate in BSE was not solely based on the concern with one's health. Rather, they were influenced by social factors. First, it depends on the social image of women's' breasts. For instance, when women's groups proposed the right to breast-feeding babies in the public, one male politician stated that: "Don't be shamed of feeding your baby in the public unless you are uncomfortable of your flat breasts. We, as men, have seen many kinds of women's breasts." Women's breasts are sexualized for male satisfaction. Thus, some women participate in BSE to escape the male doctor's gaze. Some women deny BSE because of feeling uncomfortable about exposing her breasts to other women. Secondly, doctors' work is overloaded in the current medical system; they have to see 30- 50 patients in a half-day, so each patient has only a few minutes for the examination. Some women turn to BSE due to their distrust in the doctor; even BSE is not recommended in the media.

The question of why women regard BSE as a complex issue might be related to the way women are valued in society. This is reflected in the following social factors.

#### 2-1 Taboos of uncovering one's breasts

Women do not like to talk about their breasts because it is taboo in Taiwanese society. Taiwanese women live in a society strongly influenced by a Confucian culture. They do not like to look at pictures of breasts; and when they think of breast health they think of cancer, which they do not like to talk about. In the interviews, many women expressed concern about disrobing or uncovering their breasts. The reason given was that they were struggling with what is socially acceptable. Exposure of the breasts was beset with embarrassment, sexual connotations and vulnerability. For example, one woman "felt embarrassed" because her breasts were small and flat. Another woman felt that exposure of her breast to strangers would bring her criticism, and some women might refuse to attend the BSE training because they thought the BSE procedure, perceived as having a sexual connotation, would be too uncomfortable for them.

Some researchers indicated that the association of the breast with sex and extreme modesty influences a woman's conception of breast examination [26,27]. It was noted that the presence of female nursing practitioners could decrease embarrassment. In the study, participants legitimized disrobing by convincing themselves that breast examination was a proper health-care issue, and this was further supported by the fact that a female professional would conduct the educational training.

#### 2-2 The effect of media reporting of social issues

In general, the media is the main source of healthcare information for the public [28]; use of the media has been considered a popular and effective health communication strategy [29]. The media disseminates information concerning social issues and some of that news may contain elements of a persuasive nature that prompts women to engage in BSE. For example, a news report about a woman suffering from breast cancer typically conveys a persuasive message of a cry for fear. This type of presentation acknowledges the severity of the threat, and implicitly indicates that the audience and readers are able to engage in the suggested actions. In the case of BSE, the fear appeal claims that if women do not engage in BSE, the risk of suffering from breast cancer is much greater [30]. Vellozzi et al. [31] found that the media had been successful in encouraging Hispanic women to take action to ensure breast health.

It was found that Taiwanese women's body was facing an oppressive environment due to the linkage of women's breast with sex or shame. Under the dominance of modern medical technology, women's body care is forced to be medical oriented rather than self-care oriented. Kearney [32] indicated that due to the lack of confidence to be able to detect breast cancer by themselves, some women do not practice breast self-examination to avoid the responsibility of not finding breast cancer early enough. However, Freire, in his "Pedagogy of the Oppressed," [33] argued that "no matter how she lack of knowledge or live in a silent culture, she can grasp a critical attitude to deal with her own reality through learning." In this study, we found that the decision to attend BSE was partially influenced by the inhumanity of modern medical technology.

## Conclusions

This study makes several contributions to the current knowledge about BSE. It also provides empirical data concerning BSE in practice, highlighting how the medical environment interacts with social factors and personal factors was crucial to understanding a health preventive action regarding women's attitudes towards breast cancer.

The selected group of women who attended the BSE program might have already had a positive attitude towards BSE, thus the interpretation of the findings was not able to be generalized. However, when many conflicting opinions between women's behavior and professional suggestions have been revealed, the results may assist researchers, health professionals, and the community to understand the actions of women from their perspective.

It is important to keep in mind that this study addressed the underlying considerations that prompt women to learn BSE. We recognize that how women decide to attend BSE training is influenced by personal and social factors. Therefore, it is useful to integrate the individual elements and social context in the design of a BSE program. We are convinced that the knowledge and experience of BSE practitioners is very important. Women should neither be limited by, nor entirely dependent upon, medical and technological systems when facing these problems sine alternative solutions are available. We suggest that future research should focus more on the understanding of how women weigh the risks and benefits concerning the personal and social factors, before they deny the efficacy of practicing BSE.

## Abbreviations

BSE: Breast Self-Examination; CBE: Clinical Breast-Examination.

## Competing interests

The authors declare that they have no competing interests.

## Authors' contributions

RJY conceived and carried out the study as part of her doctoral research. LHH participated in the design of the study. YSH participated in assembly of data and interpretation. UIC assisted with the study and synthesized analyses together with CSH. HDB supervised data analysis, interpretation and led the writing. All authors helped to conceptualize ideas, interpret findings, and review drafts of the manuscript.

## Appendix I: Interview guide

1. Could you tell me about your ideas and comments about BSE?

2. Why did you decide to attend the BSE training program?

What factors prompted you to learn BSE?

Probe questions:

How did you know the training program?

What did you think about this training at that time?

What were reasons for your decisions to attend this training?

3. Could you describe your feelings for performing BSE at home the first time?

4. What make it possible to perform BSE practice monthly (in regularity)?

5. What experiences did you have regarding BSE, CBE, and mammography?

## Pre-publication history

The pre-publication history for this paper can be accessed here:

http://www.biomedcentral.com/1472-6874/10/23/prepub
